# The effects of opioid policy changes on transitions from prescription opioids to heroin, fentanyl and injection drug use: a qualitative analysis

**DOI:** 10.1186/s13011-022-00480-4

**Published:** 2022-07-21

**Authors:** Julia Dickson-Gomez, Sarah Krechel, Antoinette Spector, Margaret Weeks, Jessica Ohlrich, H. Danielle Green Montaque, Jianghong Li

**Affiliations:** 1 Institute for Health and Equity, Medical College of Wisconsin, Milwaukee, USA; 2 Department of Rehabilitative Sciences and Technology, University of Wisconsin, Milwaukee, USA; 3 Institute for Community Research, Hartford, CT USA

**Keywords:** Prescription drug monitoring program (PDMP), Opioids, Prescription opioids, Heroin, Fentanyl, Opioid prescribing guidelines

## Abstract

**Background:**

Beginning in the 1990s, nonmedical use of prescription opioids (POs) became a major public health crisis. In response to rising rates of opioid dependence and fatal poisonings, measures were instituted to decrease the prescription, diversion, and nonmedical use of POs including prescription drug monitoring programs (PDMPs), pain clinic laws, prescription duration limits, disciplining doctors who prescribed an excessive number of POs, and the advent of abuse deterrent formulations of POs. This paper explores the unintended effects of these policies in the descriptions of why people who use opioids transitioned from PO to injection or heroin/fentanyl use.

**Methods:**

We conducted 148 in-depth-interviews with people who use prescription opioids nonmedically, fentanyl or heroin from a rural, urban and suburban area in three states, Connecticut, Kentucky and Wisconsin. Interviews with people who use opioids (PWUO) focused on how they initiated their opioid use and any transitions they made from PO use to heroin, fentanyl or injection drug use.

**Results:**

The majority of participants reported initiating use with POs, which they used for medical or nonmedical purposes. They described needing to take more POs or switched to heroin or fentanyl as their tolerance increased. As more policies were passed to limit opioid prescribing, participants noticed that doctors were less likely to prescribe or refill POs. This led to scarcity of POs on the street which accelerated the switch to heroin or fentanyl. These transitions likely increased risk of overdose and HIV/HCV infection.

**Conclusions:**

A careful analysis of how and why people say they transitioned from PO to heroin or fentanyl reveals many unintended harms of policy changes to prevent overprescribing and diversion. Results highlight the importance of mitigating harms that resulted from policy changes.

Beginning in the 1990s, nonmedical use of prescription opioids (POs) became a major public health crisis. Opioid prescriptions were four times higher in 2010 than in 1999 and reached 81.2 per 100 people [1]. During the same period, both admissions to emergency departments and treatment admission for prescription opioid use increased to four times the previous rates [2]. Between 1999 and 2011, overdose (OD) deaths related to opioids increased 265% among men and 400% among women [3–5]. In 2009, an estimated one in seven US residents aged 12 and older reported past nonmedical use of opioids [6].

In response to rising rates of opioid dependence and fatal poisonings, measures were instituted to decrease the prescribing, diversion, and nonmedical use of POs including prescription drug monitoring programs (PDMPs), closing “pill mills” (unethical doctors or clinics that knowingly dispensed large amounts of pain medications knowing that these medications were being used recreationally and diverted), laws limiting the duration of opioid prescriptions, disciplining doctors who prescribed an excessive number of POs, and the advent of abuse deterrent formulations of POs. Many of these began between 2000 to 2010. PDMPs for example saw a large expansion between 2000–2009 from 16 to 45 states, and another between 2010–2019 from 45–52 (49 states, District of Colombia, Guam and Puerto Rico) [7]. PDMPs have also changed over time, moving in some states from voluntary use and registration to mandatory use to anyone prescribing or dispensing controlled substances [8]. An abuse-deterrent formulation of oxycontin was released in 2010 [9]. There are currently 11 states with pill mill laws [10]; these laws prohibit pain clinics from dispensing opioids, have specific requirements for medical examinations and follow-up before and after dispensing opioids, and other requirements. Both Kentucky and Wisconsin have laws regulating pain clinics, passed in 2012 and 2016 respectively.

State PDMPs were designed to curb nonmedical opioid use and diversion by tracking scheduled medications prescribed by medical providers and dispensed by pharmacies [11]. PDMPs were predicated on the idea that reducing excessive prescribing among medical providers or overlapping opioid prescriptions from multiple providers would reduce the supply of POs available for diversion and nonmedical use [12]. While there is some evidence that PDMPs were effective in decreasing physicians’ opioid prescribing, some researchers have raised concerns that PDMPs combined with other policy changes may have had the unintended consequences of increasing rates of heroin use and overdose [11, 13–17]. In fact, some modeling studies predicted that the short-term consequences of PDMPs may be a shift to heroin and a subsequent increase in opioid overdoses, but that opioid use (including heroin use) would eventually decline as fewer people would initiate opioid use through a doctor’s prescription or from POs bought on the street [18]. However, opioid overdoses have continued to rise exponentially since 2013, surpassing 100,000 overdose deaths in 2021 [19]. Further complicating the picture, increasing numbers of PWUO have initiated their use with heroin since 2005, bypassing POs altogether [20].

While there have been many causes for the increase in overdose deaths, escalations in heroin overdoses are temporally associated with decreased opioid prescribing, lending some support to the concern that policy changes may have contributed to PO users’ switching to heroin or illicit fentanyl [21, 22]. Although the proportion of people prescribed opioids who switch to heroin appears to be small, research has found that individuals who use POs nonmedically may shift to heroin use, particularly if they inhaled or injected POs [23–33]. Studies have shown markedly increased probabilities of heroin use after nonmedical PO use compared to people who did not use POs nonmedically [27]. Among those with substance use disorders, the largest adjusted odds ratio was for people with prescription pain reliever abuse or dependence (aOR = 40.0; 95% CI = 24.6–65.3) [27].

Despite these strong temporal associations, research to determine the extent to which laws and drug reformulations to reduce the availability of POs caused the increase in heroin use are limited and findings are mixed. Some studies have found a positive association between state PDMP and heroin poisoning mortality, while others found no association [16, 34]. Other research found that the switch from PO to heroin use occurred prior to 2010 when most of the policy changes and drug reformulations occurred [21, 35–37]. Similarly, quasi experimental studies comparing overdose prior to and after reformulation of POs have found little short-term evidence that reformulation affected overdose rates beyond a shift in the types of opioids involved in overdoses [38, 39]. A longer-term analysis of the effects of reformulation found that overdose increased dramatically in areas more exposed to Oxycontin reformulation (i.e., those markets with a higher proportion of Oxycontin users) [38]. Few studies have asked people who use opioids nonmedically whether they switched to heroin and their reasons for doing so, although some qualitative research suggests that PWUD switched to heroin as rates of opioid prescribing decreased and fewer POs were available on the street [40]. In another study, abrupt discontinuation of a PO led to PWUD’s transition to heroin [41]. Reasons why POs were discontinued are relatively unexplored.

Many policy changes were implemented within a short period of time in response to increasing opioid overdoses, making the effects of such changes difficult to tease apart [42]. However, it is possible that all the policy changes mentioned have their effect on decreasing the availability and increasing the price of POs on the street. Indeed, much qualitative research has found that participants mention the greater accessibility and lower cost of heroin compared to POs as reasons for their transition to heroin [26, 30, 43–45]. While research has shown a marked decrease in availability and price of oxycontin following reformulation to abuse deterrent formulations [46], little research has examined the price and availability of other POs following PDMPs and other policy changes. However, research has found marked decreases in the price of heroin over the past two decades [36, 47]. The street price of heroin has been lower than $600 per gram every year since 2001, with costs of $465 in 2012 compared to $1237 in 1992 [47]. Unick and colleagues found that a $100 decrease in the price of a pure gram of heroin resulted in a 2.9% increase in the number of hospitalizations for heroin overdose [36]. Further, heroin has spread to regions of the US that did not formerly have heroin markets, particularly along interstate highways, although more remote, rural counties still tend to be characterized by PO use [48].

Research has also identified changing routes of administration among people who use opioids, from taking POs orally to sniffing or injecting them, or from sniffing heroin or fentanyl to injecting it [23, 33, 44, 49–52]. This transition might also have been a result of the relative scarcity and high price of POs after implementation of the PDMP and other policies, as sniffing or injection increases the efficiency of absorption, creating a more intense “high” for users. In fact, the ability to sniff or inject oxycontin and other POs was found to increase its abuse potential and led to the aforementioned policy changes and abuse deterrent formulations [53, 54]. Any increase in injection drug use can also increase the chances of drug overdoses and transmission of hepatis C virus (HCV) and HIV [55, 56].

In this paper, we use trend theory as a framework for understanding the transitions of people who use opioids from nonmedical PO use to heroin or injection drug use, and the role that changes in policies to reduce PO prescribing and diversion played in these transitions. Trend theory examines the characteristics of people who use drugs (PWUD) and historical changes, including changes in drug policy and drug distribution systems, to explain and potentially predict changes in drug use over time [57–59]. Trend theory uses a combination of in-depth qualitative research, epidemiological data and material about the historical context to explain changing drug patterns [59]. We apply trend theory to the analysis of qualitative interviews with people who use opioids (PWUO: PO nonmedically, heroin or fentanyl) in rural, urban and suburban areas of three states, Connecticut, Kentucky and Wisconsin. Reasons participants gave for transitioning from medical to nonmedical PO use, from nonmedical PO to heroin or fentanyl, and their changing routes of administration help illuminate the effects of policy change on opioid use over time.

## Methods

### Study overview

The current study is part of a larger project that aims to compare the factors that influence the effects of opioid-related laws and policies in Connecticut, Kentucky and Wisconsin on the transitions from prescription opioids to heroin, fentanyl, and/or injection drug use. An urban, suburban, and rural area was selected in each state to examine the role of the local context on these transitions. Study teams in each state conducted in-depth, semi-structured interviews with two groups: key informants and people who use heroin or prescription opioids nonmedically. The current paper draws from interviews with participants who use heroin, illicit fentanyl or PO nonmedically. Initial participants were recruited from harm reduction services or upon entry to drug treatment facilities that were identified in key informant interviews. Subsequent participants were referred to the study by PWUO who were interviewed through snowball sampling. Eligibility criteria included being 18 years or older and using prescription opioids nonmedically or using fentanyl or heroin in the past 6 months. PWUO were compensated $35 for completing in-depth interviews. We conducted 60 in-depth interviews with PWUO in Connecticut, 32 in Kentucky and 56 in Wisconsin.

Interviews with PWUO were conducted between December 2019 and August 2021 and focused on how they initiated their opioid use and any transitions they made from prescription opioid use to heroin, fentanyl or injection drug use. Participants were asked how they started PO use, whether it was prescribed to them or not, and whether their initial use was for medical or nonmedical purposes. We also asked whether they ever used heroin or fentanyl, and to describe their first use and what led to the decision to use heroin or fentanyl. We asked them to describe their routes of administration (orally, sniffing or injecting) and whether that changed over time. For those who were initially prescribed opioids by their doctors, we asked if there was ever a time when a doctor refused to write a prescription or pharmacists refused to fill it and the reasons for this. Finally, we asked participants about the relative price and availability of different kinds of PO, heroin and fentanyl on the street, whether and how price and availability have changed over time, and what they thought the reasons for these changes were.

### Data analysis

All interviews were transcribed verbatim. We used a collaborative approach for data analysis. To develop a coding tree, we selected a transcript which the multi-state research team read to develop a preliminary list of codes. The preliminary coding list was then applied to three additional transcripts—which were purposively selected to reflect different experiences (e.g., state in which the participant lived, rural or urban location)— and refined until the research team reached consensus on a final list of codes, their meanings, and the procedures for assigning them to text data. The research team then used MAXQDA software to apply the final list of codes to the transcripts. The coding was completed by six members of the multi-state research team. Coding, the development of new codes, and memoing (jottings done by coders to capture relationships between codes or initial hypotheses) were tracked by the six-person team. We used bi-weekly team meetings for troubleshooting and quality checks that included the principal investigator of the study. We also read each transcript to summarize the person’s drug use trajectory, including the drug that initiated their opioid use (particular PO, heroin), and whether they changed route of administration or drug of choice over time. These transitions were examined and compared across participants to discover patterns.

## Results

As can be seen in Table 1, a large majority of participants (134/140, 90%) started with prescription opioids, used either medically or nonmedically. Roughly half of participants started with opioids that were prescribed to them (64/134, 48%). The majority of those who started with POs (111/134, 83%) switched to heroin, fentanyl or some combination over time, although fewer than half of participants in rural Kentucky reported switching (9/20, 45%). The lower proportion of those who used heroin or fentanyl in rural Kentucky may be a result of reduced availability of heroin in these regions, although participants reported that heroin and fentanyl were widely available. Participants in this study used opioids for 17 years on average, range 1 year to 47 years, although this varied somewhat by state. Participants in CT had used the longest on average at 20 years, perhaps in part due to the long-standing heroin markets in Hartford and the greater number of participants who began their use with heroin prior to the prescription opioid crisis; KY participants used on average 18 years; and WI participants had used for 13 years on average. In general, those who reported using for over 30 years had begun with heroin. In this paper, we focus on those who initiated their opioid use with POs.

**Table 1:** Summary of participants who initiated with prescription opioids (PO) and transition to heroin/fentanyl by geographical location (*n* = 149)

		**Initiated with PO**		**Of those who initiated with PO, prescribed to them**		**Of those who initiated PO, obtained other way**		**Of those who initiated with PO, transition to heroin/fentanyl**	
	**Total**	**%**	**(n)**	**%**	**(n)**	**%**	**(n)**	**%**	**(n)**
**Connecticut**	60	82%	(50)	46%	(23)	54%	(27)	90%	(45)
Urban 20	20	95%	(19)	36%	(7)	63%	(12)	100%	(19)
Suburban 20	20	70%	(14)	50%	(7)	50%	(7)	86%	(12)
Rural 20	20	80%	(16)	53%	(9)	47%	(8)	67%	(14)
**Kentucky**	32	94%	(30)	37%	(11)	63%	(19)	63%	(19)
Urban 12	12	83%	(10)	50%	(5)	50%	(5)	100%	(10)
Rural 20	20	100%	(20)	30%	(6)	70%	(14)	45%	(9)
**Wisconsin**	56	96%	(54)	56%	(30)	44%	(24)	87%	(47)
Urban 20	20	95%	(19)	68%	(13)	32%	(6)	89%	(17)
Suburban 20	20	100%	(20)	40%	(8)	80%	(12)	90%	(18)
Rural 16	16	94%	(15)	60%	(9)	40%	(6)	80%	(12)
**Total 148**		90%	(134)	48%	(64)	52%	(70)	83%	(111)

Participants described a gradual process starting about 2010 to 2016 by which POs became more difficult to acquire as doctors became less likely to prescribe them, which in turn, led to greater scarcity and higher prices of POs on the street. These transitions occurred more or less at the same time that PDMPs and other measures occurred. Kentucky was the first of the three states to have a PDMP and was the first to move to mandatory registration and reporting in 2012, followed by Connecticut in 2015, and Wisconsin in 2017 [8]. Kentucky and Wisconsin also passed pain clinic regulations in 2012 and 2016 respectively. As can be seen in Table 2, “Dates of Transition to heroin/fentanyl from prescription opioids” the majority of participants transitioned from PO to heroin after 2010, with few transitioning before 2005.

**Table 2:**
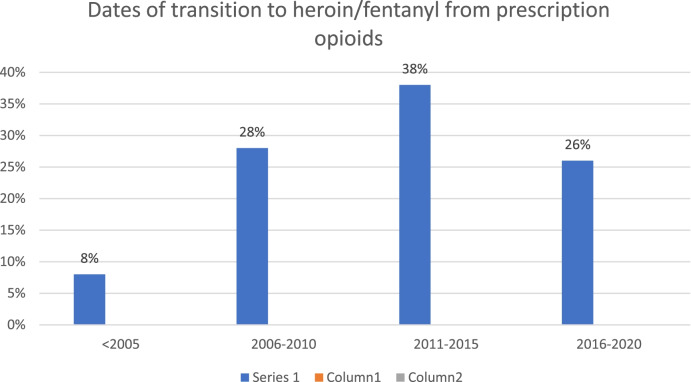
Dates of transition to heroin/fentanyl from prescription opioids

### Initiation and escalation of PO use

Participants described starting PO use for medical and nonmedical reasons and obtained their first POs through prescriptions written for them by their doctors, through friends or family members or, less frequently, by buying them illicitly.

Some participants reported being prescribed POs for long periods of time, from several months to 20 years. POs were used for legitimate pain that was caused by serious accidents or illnesses. However, over time, participants reported needing higher doses and looked outside regular prescriptions to control their pain, like the participant below who was prescribed POs for twenty years.

Participant: I’m 56 years old. I would say 20 years, I was 25 years old when I started using them because I had back issues. I started on Tylenol 3. Then… I got hurt and they put me on…Percocet. I can’t remember. There’s so many different ones I was on I can’t count…. I was on morphine, 10 milligram morphine

Interviewer: Was there times where you, they kept raising the milligrams?

Participant: They went from five, then they went up to 10 milligrams. That’s why they gave me Percocet. Then they gave me the morphine pill. I’ve been taking them for so long I started abusing it because my body was getting immune to it. I had to get more and I was running out. I had to go on the street and go buy them. It just got, over the years I spent a lot of money, thousands of dollars. I gave away jewelry for pills; whatever I can do to get a fix, put it that way, to get opiates, a pill, pain pills (Male, White, suburban WI, 56 years old)

Many participants described that the POs gave them a high in addition to controlling pain. As their tolerance increased, the pills no longer produced euphoric effects along with controlling the pain as the participant below describes.

Participant: When I first started out with it, it was I’d say either right on or maybe not enough because it was a lot of extreme pain, 9 broken ribs and a broken bone in my neck alone that just was a lot of pain. But after a few months, it started to become more of a feeling of just—the pain wasn’t there at all. If I took my medicine the way I was supposed to, I was perfectly fine, if not better than fine. And I started having that feeling, which I know now was the feeling of being high (Male, white, urban CT, 31 years old)

Many participants were surprised that they became addicted to POs and were not aware of the signs of dependence. In part, this stemmed from the ease and frequency with which doctors prescribed between the late 1990s and 2010, who no doubt believed the pharmaceutical claim that POs were not addictive if used to treat pain [60, 61]. Some participants confused withdrawal symptoms with continued pain from their injuries, as the participant below described.

Participant: I had to go through physical therapy to kind of learn how to walk again and deal with all that stuff. And after I was out of physical therapy and I’m walking again, I’m starting to feel better. It was just this feeling like I still have the pain…. So, I would take the painkiller and come to find out, I wasn’t really in that much pain. It was like normal pain or not even pain at all. It was the fact that they were lowering my dosage so really what I was feeling was the withdrawals from being on the prescription medicine so long that when they came down from 30 mg to 15 mg, it’s a big jump for the body to have been on it for 11 or 12 months, and then all of a sudden, we’re going to drop you down halfway, like cut it in half completely. So, I didn’t know that at the time. I know that now because I’ve talked to people, I’ve learned about this. Like I said, I’ve tried to go to treatment, so I know that that’s what it was. It wasn’t really pain. It was more the detox feeling (Male, white, urban CT, 31 years old).

Pain pills took away both emotional and physical pain, making them almost irresistible to participants who had been through traumatic experiences.

Participant: The first time I was introduced to pain pills was, I was 18 years old, I was in a really bad car wreck. I went through the windshield, actually, and I had seven reconstructive surgeries on my face. And it was right when Oxycontin came out, and that was supposed to be the cure all drug. You weren’t supposed to be addicted to it. Doctors were pushing it. And for about two and a half years, over my seven surgeries, I was prescribed Oxycontin 80 [mg], well, it had worked up to Oxycontin 80. It not only numbed the pain, but it numbed everything. I loved it. It became my best friend. You know, being 18 and literally not having a face, and being so uncomfortable with that, and just all the emotional pain, and physical pain, and all of that, it just numbed it all. I had found my very best friend. (Female, White, urban KY, 40 years old)

Some participants who were prescribed POs for short-term pain reported feeling high at first use and immediately sought it out to use nonmedically.

Interviewer: Describe for me the first experience using prescription opioids and what it was used for?

Participant: I believe the first time was in eighth grade, and it was because I broke my leg. And I can’t remember what it was [particular PO] but oh my God, it was great. I took one and lied to my mom. I’m like, “Hey, just leave that bottle in here, just in case it doesn’t work and I need to take another one.” And I took three or four more. And oh, I was like floating on my bed, ooh. It was just like everything was too good, too bright. Nothing mattered….

Interviewer: Did you run out of the pills before time, that first time?

Participant: Yes, I actually went to my friend because I know he had pain pills. And I told him, “Hey, I tried this. Do you want to try it with me?” And we had a sleepover and we exchanged a bunch of pain pills (White male, suburban WI, 46 years old)

Some participants described being prescribed POs that they didn’t really need or for a longer time than they needed, like the participant below who was prescribed opioids by her dentist for a tooth cleaning. In many of these cases, participants immediately felt the high of POs and were motivated to continue to use.

Participant: First time I ever used opioids for recreation, recreational uses, is I was about 19, and I had never been addicted to anything. And when I started dating this guy, and he was addicted. And he warned me. He begged me not to start, and I done it anyway. And it was a 2 milligram Lorcet. They prescribed it to me for cleaning my teeth – of all things, a tooth clean…. And I snorted it…. I really wasn't addicted to them. I was just young and stupid and trying to be part of the crowd. It was just me trying to catch up to everybody else, I guess. Trying to be normal because, back then, that was the normal thing to do around here. There's nothing else to do, so everybody starts getting high. And at the time, you'd get pain pills for anything, any reason. Have you ever heard of getting your teeth cleaned and getting pain pills for getting a tooth cleaned? (White female, rural KY, 41 years old)

Because of the over-prescribing and abuse of PO, the participant above knew how POs were taken recreationally and sniffed it at first use.

Other participants described using POs medically but never received a prescription. Often, participants were given opioids by friends, partners or family members. The woman below borrowed pain pills from her boyfriend after cutting her finger in a work accident because she didn’t have insurance.

Participant: I was in my early 20s… I was working in a kitchen. It was my first real job as a cook outside of school… I ended up cutting the tip of my finger off while chopping up bacon my second week in… I didn’t have insurance at the time, so they wouldn’t, it wasn’t worth even prescribing me, so they just didn’t because I couldn’t afford it. And my boyfriend at the time… he threw out his back and he ended up getting a bunch of medication for it, muscle relaxers and Vicodin. He had a severe addiction to opioids at the time that I was aware of, but not the full extent until much later. So no, he didn’t want the Vicodin. He told me it was because he just wants to stay away from it, blah, blah, blah. But it turns out later, the real reason, they weren’t strong enough for him. So, he gave them to me to hold onto right around the same time I hurt myself, and I’m like, “Okay, he’s not gonna take them, I might just have one here, just see what it is.” The first time taking it, oh my gosh, all the pain went away. (White female, suburban WI, 23 years old).

Medical use without a prescription was facilitated by the large number of POs being prescribed with easy access to left-over medications. This also facilitated nonmedical use, as some participants described initiating opioid use as adolescents from family members’ supplies or from friends who had taken family members’ prescriptions.

Participant: Pain pills were never hard to find at the time. Even being young and being in school, all my friends knew somebody who could get them, or I had friends that stole them from their family medicine cabinet. I had friends that had older people that sold them the pills. I had a cousin who had access to, God, I don't even know. (White male, urban KY, 25 years old)

Importantly, the participant above states that POs were still easy to obtain up to 2012 when the Kentucky PDMP switched to mandatory use by prescribers and dispensers.

Less frequently, participants initiated opioid use from POs they bought on the street. Again, this was a function of the easy access to POs that came with over prescribing.

Participant: Well, first time I ever used prescription opioids, I was 15. I got them off the street, basically – back then, you could…. It was Lorcet…. It was on the street down here in town, on the street – back then, you could run around on the street and do pretty much anything. (White female, rural KY, 42 years old).

### Increased tolerance

Almost all participants reported a time in which they needed to take more POs to control their pain or to get high. All described taking POs to avoid withdrawal. While some continued to get POs from their doctors, others used more than what their physicians prescribed. Obtaining more could be accomplished through prescriptions from pill mills, doctor shopping or finding “crooked” doctors who were willing to prescribe large quantities of POs. Those who went to pill mills or doctors, in turn, sold POs to other people and on the illicit market.

Participant: They called it the pill pipeline, people were driving to Florida, getting prescriptions and then driving back with them. They were the people that were basically taking people, they were giving them their money and having them go up, get their prescriptions and then they got the packs…. So, they had a little business going on for a little while. (White female, urban KY, 30 years old).

Interviewer: So, when you first tried it, how did you get it?

Participant: I got it through one of my friends. He had a dirty doctor. He got caught [the doctor]. He was just laying out, you know, here’s a couple hundred dollars, and he would write you a prescription for whatever. Anyway, I was buying them from my buddy that was getting them from him. (White male, suburban WI, 34 years old)

More often, participants reported buying POs on the street. As this participant describes, supplementing their legitimately prescribed POs with those bought on the street often led to increased tolerance as participants were limited in what they were able to buy to what was available on the street, which often was a higher dosage or different type of opioid.

Participant: That’s what sucks, is like I’m only on 10 mg oxycodone, and only 30 mg morphine. In the beginning I got, I think it was 90 morphine. But I have weaned myself down on that, and my doctor’s helping me wean down on oxycodone, which is good. But anytime I run out, I can’t find little 10 oxycodone that I’m on and that’s the thing. I would always get like a 30 mg or something, and I’d try to cut that in half, whatever. Because every time that I end up taking more than I’m prescribed, then I’m addicted more. And when I get my normal script back from my normal doctor, it’s not enough. (White male, suburban WI, 43 years old.)

Increased tolerance was particularly true for participants who used heroin to supplement their medical or nonmedical use, as heroin quality and strength varied considerably as will be described in more detail below.

### Changing landscape: PDMP, pill mills and prescribing practices

While some participants weren’t aware of changes in laws or policies and simply noticed that POs on the street were becoming scarcer over time, others directly experienced some of the changes and their effects on PO availability and price. One such change was the closing of “pill mills” that were a source of some POs sold illicitly in Kentucky and Connecticut.

Interviewer: What have you heard about legal efforts to limit prescription opioid supplies?

Participant: I was just watching the news on that. Anywhere, you have to be on your death bed to get pain pills. Like I said, in Florida, everybody got busted.

Interviewer: What would people go there to get?

Participant: Anything and everything. The oxycontin, the Lortabs, everything for pain. They would get somebody’s address in Florida or something and go to the doctors. And they would give them a 90-day prescription. Somebody would pay for the trip and give them so many pills. And they’d get the rest of them or something. Everybody, the doctors, and everybody got busted in Florida. And people that were doing it got busted. That’s why I thought everybody couldn’t find pills anymore. (White Female, urban KY, 53 years old).

Others noticed that doctors were no longer prescribing opioids which they attributed to increased media coverage of PO abuse, disciplining physicians who over-prescribed and the PDMP.

Participant: Say, I got kidney stones or something or anyway that you know how to do it to get it. But now they’re really strict. Now, with the computer situation everybody knows everything. Every doctor you go see, they know what you’re on. I don’t care what doctor you see now, they know. Back in the day, you can go to three different doctors and get three different medications and they don’t know. But now with the computer age, it’s everywhere. (White male, suburban WI, 53 years old).

Many states’ PDMPs require prescribers and dispensers to report opioid prescriptions to the PDMP immediately, which is then available for everyone with access to the database, including everyone who has registered (physicians, APN, PA and pharmacists in the three states studied here). This makes it easy for prescribers to check whether participants have been “doctor shopping” and have received prescriptions from more than one doctor.

Many participants who had been prescribed opioids for long-term pain were cut-off by providers after the changes in law, often abruptly, with little consideration for tapering or prescribing MOUD or referring patients to drug treatment. The participant below describes how his doctor made him take urine drug screens after repeatedly trying to fill his prescription early. After finding a medication in his urine drug screen that he wasn’t prescribed, he was cut-off with a very abrupt taper.

Participant: Well, no, she found a medicine in my system that wasn’t supposed to be in a drug test and right then and there, she was like, “Okay, we’re going to start weaning you off of it.“ And it literally was within a three month period that I went from taking I don’t know, 150 mg of Percocet a day…10 mg of Valium and maybe 15-20 mg of Vicodin to taking 75 mg of Percocet, 5 mg of Valium, everything was cut directly in half. And then the very next month, it was cut in half one more time and the very next month, there was absolutely nothing. And I’ve talked to so many clinicians and many people and treatment centers that told me that was just completely unsafe to wean somebody off that had been on painkillers and that high of a dosage for two years to just wean them off in three months and then expect there to be no habit or no repercussions. So, I’m not saying it’s all her fault. It’s partially the system’s fault. It’s my fault for going and buying what I was buying and abusing other drugs, but it definitely could’ve been done a different way. (White male, urban CT, 31 years old)

Another participant who had been prescribed high doses of POs for over a year was cut off abruptly after he started taking buprenorphine to manage his withdrawal symptoms as he was being tapered off opioids by his doctor. When the doctor found out that he had taken buprenorphine, he was immediately taken off POs without referrals to drug treatment.

Participant: They started to [taper me] a little and then they kicked me off because I went and got some Suboxone [buprenorphine] one day 'cause I was really sick, so I went to detox for a couple days and then I – so I could lower my dose, so I wouldn't be so sick and then I went back and got pills for a week and then the doctor found out I went there and she cut me off. I said but you’ve been giving me them…. I'm addicted to these things….so I had no choice but things I didn’t want to do….

Interviewer: So, they just kind of found out that you got on the Suboxone?

Participant: Well I told’m [that I took buprenorphine] and I said you can’t just cut somebody off like that. It made me sick. You can’t do that to a man. They pushed me down the road to dope. Like I said, I wasn’t selling them for dope but I did a little bit just to stretch it and then they pushed me to it really…

Interviewer: Yeah. So, did they—did your doctor provide or recommend any alternative for you when they cut off your prescription?

Participant: No. She’s like, “Go back to where you got the Suboxone,” being a smartass. That’s all she could say. I said I’m gonna be wicked sick. I said this is messed up and they didn’t care (White male, rural CT, 45 years old).

Another participant talked to a doctor about her concerns that her PO use was becoming problematic. The doctor cut her prescription immediately without tapering or referral to MOUD or a drug treatment program.

Participant: At the age of 25, I want to say it was 25, 26, I actually came out and told my doctors that I was addicted to the pills and I was abusing them. They took me off, weaned me off and transitioned me to just doing Tylenol and Motrin.

Interviewer: From Vicodin to Motrin and Tylenol?

Participant: From Percocet at that point… Yeah, because I told them I had a problem with it. But I found myself still withdrawing from them because I had taken them for so long. I couldn’t sleep or anything, so I started buying on the street (Hispanic female, urban CT, 33 years old.)

Only one participant in our study had a doctor ask if they wanted to go to a drug treatment program after refusing to prescribe an opioid.

Participants also reported that doctors wouldn’t prescribe opioids to them even for acute pain incidents because the patient’s medical charts indicated they had been prescribed MOUD or that they had an OUD. This occurred even in cases in which the source of pain was easily observable, and POs were customarily prescribed, like a broken arm, stab wounds or post-surgery.

Interviewer: Have you ever been denied a prescription opioid?

Participant: Oh yeah, absolutely. Since I’ve gotten off them and I’ve been to the hospital a few times with some pretty bad injuries… Once it’s on file that I’ve been to treatment centers and I’m considered an addict or I have been, whatever, they’re not giving me nothing. Here take a Tylenol. I hope you feel better. It’s like I have two cracked ribs, I don’t’ think Tylenol 3 is gonna do it but they don’t want to give it you because you’re on file as abusing pain medication so that’s definitely happened to me a few times since…. And it’s like, to me that’s not right. If there’s a legitimate problem, then there’s a legitimate problem. I can understand if someone’s coming in off the streets complaining of neck pain, you do an x-ray and there’s nothing on the x-ray, yeah okay. If there is something wrong, I feel like you should treat it regardless if the person has a past of opioid addiction or abuse because the bottom line is, it’s one of very few things that helps that type of pain unless they figure out something that takes that pain away (White male, urban CT, 31 years old)

Doctors sometimes refused to prescribe opioids if participants had been on MOUD even if they were no longer taking it. In other cases, they told patients that they were unable to prescribe opioids or other drugs that cause respiratory depression like gabapentin or benzodiazepines when a participant was on MOUD because it was contraindicated.

## Fewer prescriptions on the street: transitions of route of administration and switches to heroin

Much qualitative research has found that PWUO reported switching from nonmedical PO use to heroin because it is cheaper and more accessible [44]. Similarly, qualitative research has demonstrated that PWUO often switch from taking POs orally to sniffing or injecting them, as this gives them an increased high [44]. Few studies, however, have shown how changes to reduce the prescribing of opioids led to scarcity on the street and increased price. It is in this context that many participants in our study reported switching to heroin or changing their route of administration.

Participant: So, one of my friends who had been helping me to get painkillers, he had been using for a little while and he had already talked to me about it a few times, like you really should stop wasting all your money buying these pills. You’re running out of money and after a month or two when I really noticed that like my money was getting really low… and we wanted to go meet somebody for pills. The person didn’t show up, something happened, and my friend was just in my ear. So we jumped on the bus, we came up here, he brought me somewhere, grabbed it [heroin] and after that it was just all heroin because it was just so much cheaper and it had the same effect that painkillers had, if not stronger, more intense in the beginning, if anything, so I just didn’t care and that was it (White male, urban CT, 31 years old).

The participant above went on to describe that when he first used heroin, it was $3 a bag, which was equivalent to a 10 mg Percocet that would sell for $10.

Some participants described the increase in price as directly related to the scarcity of POs after changes in prescribing practices.

Participant: I’ve noticed that prescription opioids have skyrocketed all the way up to $2 a milligram. And since I’ve first tried heroin to present day, I think that, because I paid $40 the first time… I’ve only really seen or heard of that same amount going for around $60, tops, and it’s rare. It’s usually for $50. So, really, it only jumps like $10 in the last 10 years. With opiates, that’s like doubled in price for the pills.

Interviewer: Right. Okay. Do you attribute that to the new laws, and policies, and stuff, that have been put in place?

Participant: Yeah, like they’re cracking down, so people are more apprehensive to sell them if they’ve got them. And then, of course, you run into the scenarios where, you know, how bad do you need it? How much money do you got? Highest bidder kind of thing. And I’ve seen 60 milligram OxyContin go for $250. So, there’s those rare occasions where there’s a bidding war over the last available, and it gets pretty colorful. (White male, rural WI, 36 years old)

Participants described that using heroin created a vicious cycle of increased tolerance because it was stronger, and the quality could be variable. Similarly, injecting was described as creating a more intense high and was thought to increase tolerance and addiction.

Participant: So then we’d end up buying heroin because it’s so much cheaper than pills. You can get a big bag of it, and it does more. And then, you snort it. Well, I was satisfied. I was happy. But I hated it because every month, then I’d have to go back to my normal pills after a week of doing heroin. And then, I’d be at a different level. So, then when I do my regular pills, it’s not enough. So, I would feel horrible. You have to do a certain amount just to feel not sick anymore. But everyone does a little more than that. And that’s why your tolerance keeps going up. (White male, suburban WI, 46 years old)

While most participants in the face of withdrawal or not being able to find POs described transitioning to heroin or injection or both, others decided not to transition fearing that they might die from overdose, or “never come back” from addiction, like the participant below, who would not transition to heroin.

Participant: Well, I mean, I’ve had – when I went to get something that I wanted and they didn’t have it, they’d say, “Well, no, we have heroin. Do you want to try it? It’s real good. It gives you a good buzz.” I – “No, I don’t because I’ve had a few friends overdose on heroin. I’ve had a few friends die from overdosing on heroin and I just don’t want to go there.” That’s one I don’t want to – I would never try it. (White female, rural KY, 53 years old)

## Discussion

**Fig. 1:**
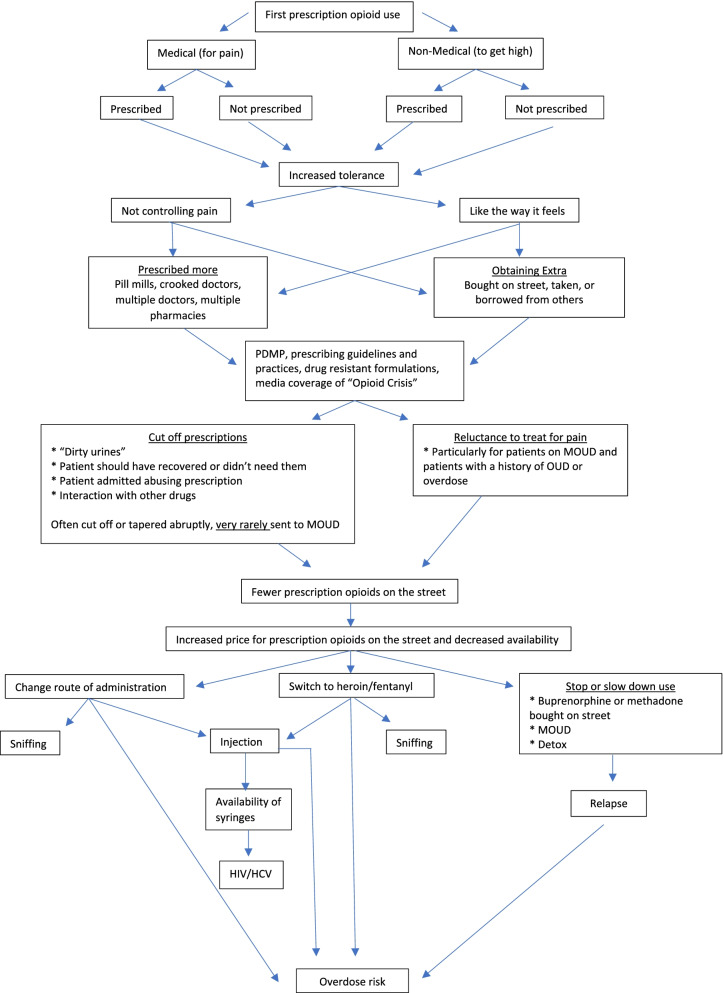
Transitions from prescription opioid use to injection and heroin/fentanyl use

Results from this study provide evidence of unintended consequences of state PDMPs and changes in opioid prescription guidelines. Figure 1 shows a sequential model with key transitions and ways that the PDMP, abuse deterrent formulations and other laws and policies affected these transitions to nonmedical use, different modes of ingestion and heroin/fentanyl. As mentioned, most participants initiated opioid use with POs, not heroin or fentanyl. These POs were either for a medical (pain) or nonmedical reason (recreation/to get high) and could have been prescribed to them or not. Some participants reported being prescribed POs for several months or years. Others described immediately seeking out more opioids for a nonmedical reason after taking an opioid for the first time. Use increased with increased tolerance because their current dose did not control the pain, or because they “liked the way prescription opioids made [them] feel.” In such cases, participants described seeking out more opioids either through being prescribed more, obtaining opioids from friends or family members who had them, or buying them on the street. Some participants were prescribed higher doses or more pills from their regular doctors over time. Others described doctor shopping to obtain prescriptions and using multiple pharmacies to fill them. Still others went to “pill mills” in Florida or found local “crooked doctors” who were willing to prescribe large quantities of opioids. Buying POs in the street or using heroin to supplement POs that were prescribed to them created a vicious cycle for participants, as they often had to take what was available, which could be POs of different doses or strength, or heroin or fentanyl, which could vary in purity.

The PDMP, new prescribing guidelines, drug resistant formulations, and increased media coverage gradually changed the ease with which participants could obtain POs from their doctors and the availability of POs on the illicit drug market. Participants reported that this was a process that started around 2010 until 2016 rather than a sharp divide between before and after implementation of these policies, perhaps reflecting media coverage and increased concern over nonmedical PO use before policy changes, and delays in when PDMPs were implemented. PDMPs have also changed over time, often moving from more voluntary to mandatory use [62]. Interviews with people in charge of developing and implementing the PDMP in each of our study states talked about changes over time moving from encouraging physicians and prescribers to register with the PDMP to mandatory checking of the PDMP when prescribing or dispensing any opioid [63]. PWUO reported changes in physicians’ prescribing practices that occurred gradually over time, centering around the time PO restriction policies were initiated. Many participants reported that their physicians cut off their prescriptions, often abruptly, for any of the following reasons: the patient had other drugs in their urine drug screens; the acute need for the prescription ended and doctors felt that participants no longer needed POs; the participant reported to the doctor that they were becoming addicted to the PO; or the doctor cut the prescription because of interactions with other drugs, particularly benzodiazepines. Participants also reported that doctors were less willing to prescribe POs to treat pain, particularly for patients with documented opioid use disorders (OUD) or patients on medications to treat opioid use disorder (MOUD), i.e., buprenorphine or methadone. These prescribing practices led to fewer POs available “on the street”, i.e., in the illicit market which, in turn, led to higher prices. In response, participants reported changing their routes of administration to sniffing or injecting POs, which allows more efficient absorption, switching to heroin or fentanyl, which was cheaper and more accessible than POs, or stopping or slowing down their opioid use. Sniffing and injection increases risk of overdose, while injection also increases risk for infectious diseases like HIV and HCV. Some participants reported going to detox to slow down their opioid use, buying buprenorphine or methadone on the street, or entering MOUD treatment. However, after a period of time in treatment and particularly after detox, if relapse occurs it carries a heightened risk of overdose.

The PDMP and other policy changes that came about to decrease the overprescribing of opioids in the 1990s and early 2000s are historical events that changed the course of the opioid epidemic, albeit in sometimes unintended and harmful ways. Rather than decreasing opioid use, these policies instead appear to have caused at least some people who were initially prescribed opioids to transition to riskier drug use such as injection or using heroin/fentanyl. Although our participants do not represent the majority who were prescribed opioids for pain, most of whom take them only as prescribed and do not switch to heroin, the increasing rates of overdose deaths due to heroin and fentanyl suggests that the number of people who switched to heroin is not trivial. The existing market for POs on the street was replaced with heroin when POs became less available [64]. Trend theory is a useful framework for understanding these changes over time [59]. Importantly, while much policy research measures changes after implementation of the policy, change can be more gradual than usually assumed in such analyses.

It would be difficult and undesirable to eliminate the PDMP and other measures that are now in place to prevent over or inappropriate prescribing of opioids. However, a number of steps can be taken to mitigate the negative effects of this change that we now see. There is a need for best practices for patients who use opioids nonmedically, become physically dependent or develop an opioid use disorder (OUD). These guidelines could include screening tools to assess whether patients who were prescribed opioids have developed OUD who can then be offered medications to treat opioid use disorder (MOUD). Abrupt discontinuation of prescriptions for patients who appear to use POs nonmedically by refilling too early or taking other opioids bought on the street can cause harm as patients may seek alternatives on the street. Physicians discontinued or tapered PO abruptly when they found opioids other than those that were prescribed in participants’ urine drug screens or when a participant told her doctor she was misusing her medication. Such occasions are ideal opportunities to assess a patient’s problematic substance use and intervene with MOUD. In contrast, abrupt continuation can damage the patient/doctor relationship and is likely to discourage disclosure of substance use. It is especially distressing that a participant who took buprenorphine to manage his withdrawal symptoms as he was tapered from long-term opioid use by his doctor was cut off his opioids even more quickly for doing so. More consistent use of MOUD during discontinuation of POs may be necessary for patients who have been on them for a long time or who report nonmedical use.

There is also a need to re-examine the treatment of pain in light of the opioid crisis. While long-term opioid use to treat chronic pain shows little evidence of effectiveness, POs are still recommended for acute pain such as post-surgery [65, 66]. In our study, many patients were denied medications to manage acute pain after having been diagnosed with an OUD or taking MOUD. Such actions are punitive and harmful as many patients may seek relief from pain using heroin or fentanyl. Further, these punitive actions are stigmatizing to people who use opioids as they suggest that people with opioid use disorder are untrustworthy or undeserving of relief.

The under-treatment of pain for people on MOUD or with OUD is common and stems from four medical misconceptions [67]. The first misconception is that MOUD on its own is sufficient to control pain. People on MOUD do not receive sustained analgesia and, in fact, long term opioid use including MOUD use is likely to make patients more sensitive to pain both due to increased tolerance to opioids and opioid-induced hyperalgesia [68]. In such cases, patients are likely to perceive higher levels of pain and may need additional support to monitor their acute pain. Second, many clinicians fear that use of opioids for analgesia may trigger relapse although there is little evidence that this, in fact, occurs. There is more evidence to suggest that untreated pain and the emotional distress it causes may trigger relapse [69]. The third misconception is that opioid analgesia and MOUD may cause respiratory depression. However, tolerance to the respiratory effects of MOUD occurs rapidly and reliably and the risk of additive effects of opioid analgesia and MOUD has not been clinically demonstrated [67]. Finally, physicians may believe that reporting pain may be a manipulation to obtain opioid medications, or drug seeking because of opioid addiction. MOUD by definition will decrease the euphoric effects of opioids; thus, drug seeking is unlikely to be a reason for reporting pain if a patient is on MOUD. Treating pain for a patient taking MOUD or who is actively using illicit opioids is challenging but necessary [70–72]. Current guidelines are based on expert opinion as few clinical trials have been conducted on acute pain management for people with OUD or on MOUD to date [70]. However, guidelines recommend continuing MOUD and treating pain with higher doses of opioids, other non-opioid analgesics and, for buprenorphine, splitting the daily dose of buprenorphine [67, 70].

It is estimated that less than 20% of people with OUD receive MOUD [73–75]. Research suggests that the low uptake of MOUD is driven by stigma [76]. There is great need to educate physicians, people who use opioids, and the community at large about the safety and efficacy of MOUD, and the dangers of detoxification and other contraindicated “drug treatments” that may decrease tolerance and increase overdose risk. There is also a need to increase community education about nonmedical opioid use, overdose and its reversal and the benefits of harm reduction.

A careful analysis of how and why people say they transitioned from PO to heroin or fentanyl reveals many unintended harms of policy changes to prevent overprescribing and diversion. They also reveal ways we can mitigate harms that have already occurred and treat people who use opioids with compassion and respect, not stigma and punishment.

## Data Availability

Data is available from the first author upon request.

## Abbreviations

HCV: Hepatitis C virus
HIV: Human Immunodeficiency virus
MOUD: Medications to treat opioid use disorder
OUD: Opioid use disorder
PDMP: Prescription drug monitoring programs
PO: Prescription Opioids
PWUD: People who use drugs
SUD: Substance use disorder
